# Targeting the Toll-Like Receptor 4 Ameliorates Heart Failure in Aged Mice by Inhibiting the Formation of Neutrophil Extracellular Traps

**DOI:** 10.33549/physiolres.935671

**Published:** 2026-02-01

**Authors:** Huan LIU, Xiangdong CHEN, Zhilin WU

**Affiliations:** 1 Department of Anesthesiology, Union Hospital, Tongji Medical College, Huazhong University of Science and Technology, Wuhan, China; 2 Institute of Anesthesia and Critical Care Medicine, Union Hospital, Tongji Medical College, Huazhong University of Science and Technology, Wuhan, China; 3 Key Laboratory of Anesthesiology and Resuscitation (Huazhong University of Science and Technology), Ministry of Education, Wuhan, China

**Keywords:** Heart failure, Cardiac function, Inflammation, Toll-like receptor 4, Neutrophil extracellular traps

## Abstract

Heart failure (HF) is a prevalent cardiovascular condition among the elderly population, with an incidence rate that continues to rise annually, highlighting the urgent need for effective therapeutic interventions. Sustained activation of Toll-like receptor 4 (TLR4) may contribute to left ventricular dysfunction and adverse cardiac remodeling through the induction of myocardial inflammation and oxidative stress – pathological processes that closely align with the hallmark features of HF. Preclinical studies in animal models have demonstrated that TLR4 deficiency improves cardiac function in aged mice; however, the precise role and underlying mechanisms of TLR4 in human HF remain poorly understood. This study aims to test the central hypothesis that TLR4 serves as a critical molecular link between chronic inflammation and the pathophysiology of HF. HF was induced in 18-month-old male C57BL/6J mice *via* continuous subcutaneous infusion of isoproterenol (ISO, 30 mg/kg/day) over a period of 3 weeks. Thereafter, mice received daily intraperitoneal injections of the TLR4 inhibitor TAK-242 (2 mg/kg), deoxyribonuclease I (DNase I, 5 mg/kg), or the peptidylarginine deiminase 4 (PAD4) inhibitor GSK484 (4 mg/kg) for 7 consecutive days. Cardiac function was assessed using a ultrasound imaging system. HE staining and Masson staining were employed to evaluate myocardial pathological changes and collagen deposition. ELISA was performed to measure serum levels of myeloperoxidase-DNA (MPO-DNA), neutrophil elastase-DNA (NE-DNA), cTnI, NT-proBNP, IL-1β, IL-6 and TNF-α. Immunofluorescence staining was performed to detect the co-localization levels of Ly6G with myeloperoxidase (MPO) and citrullinated histone H3 (cit-H3) in myocardial tissue, in order to assess the formation level of neutrophil extracellular traps (NETs). Western blot were utilized to determine the expression level of TLR4 protein. The expression of TLR4 was significantly upregulated in the myocardial tissue of aged HF mice. Inhibition of TLR4 not only markedly improved cardiac function but also alleviated pathological damage to myocardial tissue and reduced collagen fiber deposition. Concurrently, it also decreased the serum levels of MPO-DNA, NE-DNA, NT-proBNP, cTnI, and inflammatory factors. Moreover, the colocalization levels of Ly6G with MPO or cit-H3 in myocardial tissue was also diminished. These findings were consistent with the effects observed following DNase I and GSK484 interventions. Targeting TLR4 can mitigate inflammatory responses and enhance cardiac function in HF mice by inhibiting NETs formation.

## Introduction

Heart failure (HF) as one of the leading causes of mortality in the elderly, has garnered significant attention from the global public health community. The age-related degenerative changes in the myocardium and vascular system, coupled with pathophysiological alterations such as chronic inflammatory activation, oxidative stress injury, and metabolic homeostasis imbalance, synergistically interact with the pathological progression of HF, thereby promoting its onset and development [1]. This results in symptoms such as dyspnea, progressive decline in exercise tolerance, and chest tightness. Several cases may lead to prolonged bed rest and cognitive or emotional disturbances [2]. Research indicates that chronic low-grade inflammation serves as a central driving force for cardiovascular disease in the elderly, with its progression closely linked to age-associated immune system homeostasis imbalance [3]. Chronic systemic inflammation accelerates cardiac decompensation in elderly patients by mediating dysfunction in cardiomyocytes and cardiac microvascular endothelial cells [4]. Therefore, it can be concluded that persistent inflammation induced by immune dysregulation plays a critical role in cardiac insufficiency and represents one of the key mechanisms underlying senile HF.

The occurrence and progression of HF are closely associated with inflammatory responses and immune dysfunction. Toll-like receptor 4 (TLR4), a pivotal pattern recognition receptor in the innate immune system, activates downstream immune cascades by recognizing pathogen-associated molecular patterns (PAMPs) and damage-associated molecular patterns (DAMPs) [5]. In recent years, the significance of TLR4 in cardiovascular diseases, particularly in senile HF, has gained increasing attention [6,7]. Our previous research demonstrated that TLR4 expression is significantly upregulated in elderly patients with HF. Inhibition of TLR4 expression markedly enhances insulin sensitivity, alleviates inflammatory responses and oxidative stress in the cardiovascular system, and consequently improves cardiac function in the elderly [8]. Furthermore, TLR4 knockout in aged mice resulted in reduced dendritic cell maturation, activation, and antigen presentation, accompanied by diminished inflammation and significant improvement in coronary atherosclerosis, underscoring the crucial role of TLR4 in regulating cardiac function [9]. Emerging studies also indicate that TLR4-mediated immunothrombosis plays a critical role in coronary artery disease, impacting cardiac microcirculation and exacerbating the progression of senile HF [10,11]. These findings not only deepen our understanding of the regulatory network of TLR4 in elderly HF but also provide a theoretical foundation for precise intervention strategies targeting the TLR4 pathway. Nevertheless, the specific regulatory mechanisms of TLR4 in the pathogenesis of HF require further investigation to offer more robust mechanistic evidence for the clinical application of TLR4 inhibitors.

Neutrophils serve as the core effector cells of the innate immune system, characterized by their bidirectional polarization into the N1 pro-inflammatory phenotype and the N2 repair phenotype. Their phenotypic switching is regulated by microenvironmental signals [12]. In infectious and inflammatory microenvironments, N1-polarized neutrophils are more prone to release neutrophil extracellular traps (NETs), which consist of a network structure formed by depolymerized chromatin scaffolds and antimicrobial proteins, effectively capturing and killing pathogens [13,14]. Studies have demonstrated that TLR4 activation serves as a critical mechanism driving the polarization of neutrophils toward the N1 type and promoting excessive NETs formation [15,16]. A study by Carminita *et al.* highlighted that NETs abnormally accumulate during myocardial ischemia and injury, positively correlating with the severity of cardiovascular diseases [17]. Notably, in elderly individuals, the imbalance of neutrophil homeostasis and abnormal NETs release are significantly associated with ventricular remodeling progression and systolic function deterioration, leading to microcirculatory disturbances and myocardial injury [18].

Based on the accumulated evidence, we propose the following central hypothesis: in age-related HF, TLR4 activation promotes neutrophil polarization toward the N1 phenotype, leading to excessive NETs release, which subsequently amplifies myocardial inflammation, induces microcirculatory dysfunction, and accelerates fibrotic remodelling – collectively contributing to progressive cardiac dysfunction. This study aims to validate this signaling axis in an isoproterenol (ISO)-induced HF mouse model using aged animals, thereby providing a mechanistic and theoretical foundation for immunomodulatory strategies targeting the TLR4-NETs pathway.

## Materials and Methods

### Materials

ISO and deoxyribonuclease I (DNase I) were purchased from Merck (Darmstadt, Germany). TLR4 inhibitor TAK-242 and peptidylarginine deiminase 4 (PAD4) inhibitor GSK 484 were purchased from MedChemExpress (Monmouth Junction, NJ, USA). Anti-TLR4 antibody, anti-myeloperoxidase (MPO) antibody, anti-citrullinated histone H3 (cit-H3) antibody, and anti-GAPDH antibody were purchased from Abcam (Cambridge, UK). Anti-lymphocyte antigen 6 complex G site (Ly6G) antibody was purchased from Cell Signaling Technology (Danvers, MA, USA). Enzyme-linked immunosorbent assay (ELISA) kits for MPO-DNA, neutrophil elastase-DNA complex (NE-DNA), N-terminal pro-B-type natriuretic peptide (NT-proBNP), interleukin (IL)-1β, IL-6, and tumor necrosis factor-α (TNF-α) were purchased from Saipei (Wuhan, China). Mouse cardiac troponin I (cTnI) ELISA kit was purchased from Huamei (Wuhan, China). Hematoxylin-eosin (HE) staining kit and Masson trichrome staining kit were purchased from Servicebio (Wuhan, China).

### Animals and grouping

Aged male C57BL/6J mice (18 months old, 30~35 g) were provided by Animal Experiment Center of China Three Gorges University and maintained under specific pathogen-free (SPF) conditions with a controlled ambient temperature of 22–24 °C and relative humidity of 40–70 %, with *ad libitum* access to water and standard chow. An ALZET 1004 osmotic micro-pump (Alzet, California, USA), equipped with a 100 μl reservoir, was subcutaneously implanted into the backs of the mice. The HF mouse model was induced *via* continuous infusion of ISO (30 mg/kg/day) [19]. (1) To examine the effect of ISO on TLR4 protein expression in the myocardial tissue of aged mice, ISO was administered *via* continuous infusion for durations of one week, two weeks, and three weeks, designated as the ISO-1W, ISO-2W, and ISO-3W groups, respectively. Additionally, a control (Con) group received an equivalent volume of vehicle. Each group consisted of 8 mice. (2) To investigate the effects and underlying mechanisms of TLR4 inhibition on myocardial injury in HF mice, HF models were established through continuous ISO infusion over a period of three weeks. Two weeks after ISO infusion, mice were intraperitoneally injected with either 2 mg/kg TAK-242 [20], 5 mg/kg DNase I [21], or 4 mg/kg GSK484 [22], once daily for 7 days. These groups were labeled as the TAK-242 group, HF+TAK-242 group, HF+DNase I group, and HF+GSK484 group. The Con and HF groups received an equivalent volume of vehicle, with 8 mice per group. This study was approved by the Institutional Animal Care and Use Committee of Huazhong University of Science and Technology ([2024] IACUC Number: 4865), in accordance with the ethical standards of the Declaration of Helsinki.

### Echocardiography

Mice were anesthetized *via* intraperitoneal injection with a 0.3 % sodium pentobarbital solution at a dose of 30 mg/kg body weight. Subsequently, echocardiographic assessments were conducted using a color doppler ultrasound imaging system (MYLAB™ X5 VET, Esaote, Shenzhen, China). Cardiac functional parameters were recorded, including interventricular septal end-diastolic thickness (IVSd), left ventricular end-diastolic diameter (LVEDd), left ventricular end-systolic diameter (LVESd), fractional shortening (FS), and ejection fraction (EF).

### Euthanasia and organ harvesting

Following completion of echocardiographic assessment, blood was collected from the retro-orbital sinus of mice under ongoing anesthesia, and serum was separated for ELISA. Mice were then euthanized by cervical dislocation. Hearts were rapidly excised and consistently divided into two segments: the cardiac apex containing left ventricular myocardium was immediately snap-frozen in liquid nitrogen for subsequent Western blot analysis, while the mid-ventricular section – including the left ventricular wall, right ventricular wall, and interventricular septum – was fixed in 4 % paraformaldehyde for histological processing and staining with HE, Masson, and immunofluorescence.

### HE and Masson staining

Mouse myocardial tissues were fixed in 4 % paraformaldehyde at 4 °C for 24 h. Following fixation, tissues were dehydrated through a graded ethanol series (70 %, 80 %, 95 %, and 100 %), cleared in xylene, and embedded in paraffin. Paraffin-embedded blocks were sectioned continuously at 3 μm thickness using a micro-tome, and sections were mounted on polylysine-coated slides to prevent detachment. Prior to staining, sections were baked at 65 °C for 1 h to improve adhesion, then dewaxed in xylene I and II (10 min each), and rehydrated through a descending ethanol series (100 %, 95 %, 85 %, 70 %) to distilled water. For HE staining, procedures were performed according to the manufacturer’s instructions: nuclei were stained with hematoxylin for 5–10 min, differentiated with 1 % acid alcohol, blued, and cytoplasmic components were counterstained with eosin for 1–3 min. For Masson staining, key steps included staining with picro-fuchsin-acid fuchsin solution for 5–10 min, differentiation with phosphomolybdic acid, and selective staining of collagen fibers with aniline blue for 3–5 min. All stained sections were subsequently dehydrated through an ascending ethanol series, cleared in xylene, and coverslipped with neutral balsam. Histological images were acquired using a CX43 biological microscope (OLYMPUS, Japan).

### ELISA

Mouse serum samples were analyzed using a commercial ELISA kit according to the manufacturer’s instructions. Standards were serially diluted to generate a calibration curve, and both standards and test serum samples (100 μl per well) were added to pre-coated antibody microplates and incubated at 37 °C for 1 h. Following incubation, the liquid was aspirated, and each well was washed five times with 300 μl of 1× wash buffer, with thorough removal of residual liquid between washes by blotting. Subsequently, 100 μl of biotin-labeled detection antibody working solution was added to each well and incubated at 37 °C for 30 min. After washing, 100 μl of streptavidin-HRP conjugate working solution was added per well and incubated at 37 °C in the dark for 30 min. The plate was washed again, and 100 μl of TMB substrate solution was added to each well for color development, protected from light, and incubated for 10–15 min. The reaction was terminated by adding 50 μl of stop solution to each well. Absorbance was measured at the appropriate wavelength using a Multiskan FC microplate reader (Thermo Fisher Scientific, Massachusetts, USA). Concentrations of MPO-DNA, NE-DNA, NT-pro BNP, cTnI, IL-1β, IL-6, and TNF-α in serum samples were determined by interpolation from the standard curve.

### Immunofluorescence analysis

Following deparaffinization and rehydration of myocardial tissue paraffin sections, antigen retrieval was performed *via* microwave-mediated heating in sodium citrate buffer (pH 6.0). After cooling to room temperature, tissue areas were circumscribed using a hydrophobic barrier pen, and endogenous non-specific binding sites were blocked with 3 % bovine serum albumin (BSA) for 30 min at room temperature. The blocking solution was gently aspirated, and a cocktail of primary antibodies – either Ly6G (mouse monoclonal) and MPO (rabbit polyclonal) or Ly6G and cit-H3 (rabbit polyclonal) – was simultaneously applied to the sections at a dilution of 1:100. Sections were incubated overnight at 4 °C in a humidified chamber. On the following day, slides were washed three times with PBST buffer, followed by incubation with species-matched fluorescently labeled secondary antibodies (1:200 dilution) for 50 min at room temperature in the dark. After additional washing with PBST, cell nuclei were counterstained with DAPI (1 μg/ml) for 5 min. Slides were then coverslipped, and fluorescence images were acquired using a fluorescence microscope. Co-localization of Ly6G with either MPO or cit-H3 was quantified by calculating the proportion of double-positive cells to evaluate neutrophil infiltration and NETs formation.

### Western blot analysis

Fifty milligrams of mouse myocardial tissue were minced and homogenized mechanically in pre-cooled RIPA lysis buffer supplemented with 1 % PMSF protease inhibitor. The homogenate was centrifuged at 12,000 rpm and 4 °C for 15 min, and the supernatant was collected as the total protein extract. Protein concentration was determined using a BCA assay kit (Solarbio, Beijing, China), and samples were adjusted to a uniform concentration of 4 μg/μl with lysis buffer. A total of 40 μg of protein was mixed with 4× protein loading buffer and denatured by heating at 100 °C for 10 min. Proteins were separated on a 10 % SDS-PAGE gel *via* vertical electrophoresis with equal volume loaded per lane. Electrophoresis was performed at a constant voltage of 80 V during the stacking phase (approximately 30 min), followed by 120 V during the resolving phase until bromophenol blue migrated to the bottom of the gel. After electrophoresis, proteins were transferred to a PVDF membrane using wet transfer at 4 °C under a constant current of 300 mA for 90 min. The membrane was blocked with 5 % skim milk in TBST (Tris-buffered saline containing 0.1 % Tween-20) for 1 h at room temperature. Primary antibodies against TLR4 and GAPDH (1:1000 dilution) were applied and incubated overnight at 4 °C. On the following day, the membrane was washed three times with TBST and incubated with HRP-conjugated secondary antibodies (1:5000 dilution) for 1 h at room temperature. Following additional washing steps, ECL reagent was evenly applied to the protein-facing side of the membrane and allowed to react for 1 min. Chemiluminescent signals were captured using a ChemiDoc Go imaging system (Bio-Rad, California, USA). Band intensities of TLR4 and GAPDH were quantified using ImageJ software, and the ratio of target protein to internal reference GAPDH was calculated for semi-quantitative analysis.

### Statistical analysis

GraphPad Prism 8 software was utilized for statistical analysis and image generation. Data are presented as mean ± standard deviation. One-way ANOVA was performed to assess differences among multiple groups, followed by Tukey’s *post hoc* test for pairwise comparisons. A p-value of less than 0.05 was considered statistically significant.

## Results

### ISO induced TLR4 expression and NETs formation in aged mice

**Fig. 1:**
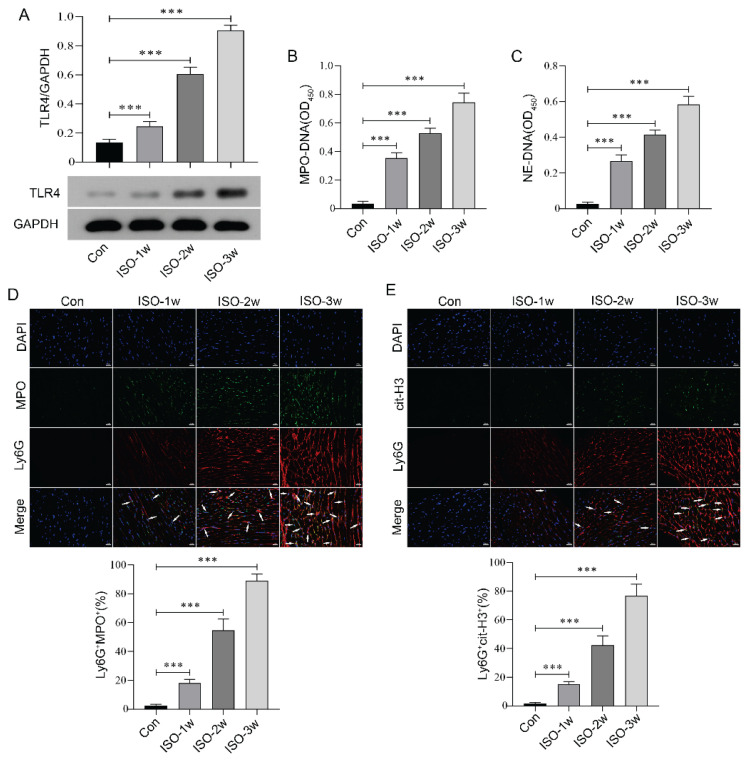
ISO induced TLR4 expression and NETs formation in aged mice. (**A**) Western blot analysis was employed to quantify the expression levels of TLR4 protein in mouse myocardial tissue at various time points following ISO induction. (**B–C**) ELISA assays were conducted to measure the concentrations of MPO-DNA and NE-DNA in the serum of mice at various time points following ISO induction. (**D–E**) Immunofluorescence staining was applied to examine the co-localization of Ly6G with MPO and Ly6G with cit-H3 in mouse myocardial tissue at various time points following ISO induction. Arrows indicate co-localized cells. Scale bar=20 μm. n=8, *** p<0.001.

As a β1-adrenergic receptor agonist, ISO induces a positive inotropic effect by continuously stimulating the β1 receptor on cardiomyocytes, leading to a marked increase in myocardial oxygen consumption and insufficient compensatory oxygen supply from the coronary arteries, which ultimately results in myocardial ischemic injury [23,24]. To investigate the changes in TLR4 protein expression during the formation of HF, we collected myocardial tissues from mice at 1 week, 2 weeks, and 3 weeks following continuous ISO infusion. Western blot analysis revealed that TLR4 expression in the myocardial tissue of aged mice gradually increased as the duration of ISO infusion extended (Fig. 1A). These findings suggest that TLR4 may play a significant role in the progression of HF.

MPO-DNA and NE-DNA represent the core components of NETs. These protein-DNA complexes not only contribute to the structural stability of NETs, but also exhibit antibacterial and pro-inflammatory properties [25]. Meanwhile, we also found that with the extension of ISO infusion time, the levels of MPO-DNA and NE-DNA in the serum of aged mice increased accordingly (Fig. 1B–C). Ly6G, a neutrophil-specific surface marker, is commonly used to identify and track neutrophils. Its co-localization with MPO and cit-H3 indicates the level of NETs [26,27]. Our results demonstrated a significant increase in the co-localization level of Ly6G with either MPO or cit-H3 in myocardial tissue as the duration of ISO infusion increased (Fig. 1D–E). Collectively, these findings suggest that the TLR4-NETs signaling axis may play a critical role in ISO-induced myocardial injury.

### Inhibition of TLR4 ameliorated heart failure in ISO-induced HF aged mice

**Fig. 2:**
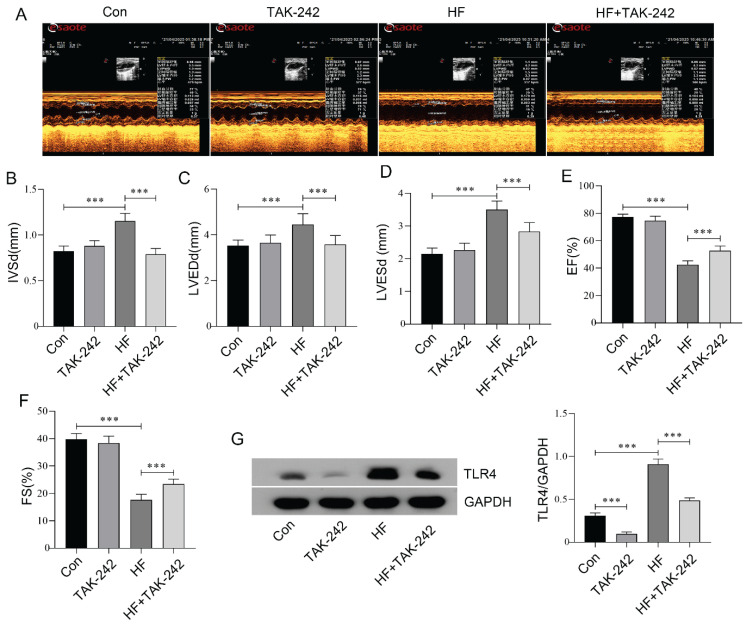
Inhibition of TLR4 ameliorated heart failure in ISO-induced HF aged mice. (**A**) A small animal ultrasound system was used to evaluate the echocardiographic parameters of HF aged mice following TLR4 inhibitor intervention. (**B–F**) Changes in cardiac ultrasonic parameters across all groups are presented. (**G**) Western blot analysis was performed to quantify the expression level of TLR4 protein in the myocardial tissue of HF aged mice following TLR4 inhibitor intervention. n=8, *** p<0.001.

HF is a clinical syndrome characterized by abnormalities in cardiac structure or function, leading to inadequate cardiac output to meet the metabolic demands of the body. Echocardiography is a widely used method for diagnosing HF clinically, and its findings play a crucial role in guiding treatment strategies [28]. To further investigate the role of TLR4 in HF, we established an HF mouse model through continuous ISO infusion over a period of three weeks. Meanwhile, we administered the TLR4 inhibitor TAK-242 and assessed cardiac function using an ultrasound imaging system. It is well known that IVSd, LVEDd, and LVESd are structural parameters used to assess ventricular hypertrophy, dilation, and remodeling, whereas FS and EF are functional parameters that reflect the systolic performance of the heart. Our results demonstrated that ISO administration increased IVSd, LVEDd, and LVESd in HF mice, while decreasing FS and EF, indicative of impaired ventricular remodeling and systolic dysfunction. Conversely, TAK-242 administration decreased IVSd, LVEDd, and LVESd in HF mice, while iecreasing FS and EF. Furthermore, TAK-242 has no significant impact on cardiac function in normal mice (Fig. 2A–F). Furthermore, our results confirmed that TAK-242 could significantly inhibit the expression of the TLR4 protein in the myocardial tissue of mice with HF (Fig. 2G). The above results suggest that inhibiting TLR4 expression can improve ISO-induced HF in aged mice.

### Inhibition of TLR4 attenuated myocardial injury in ISO-induced HF aged mice

**Fig. 3:**
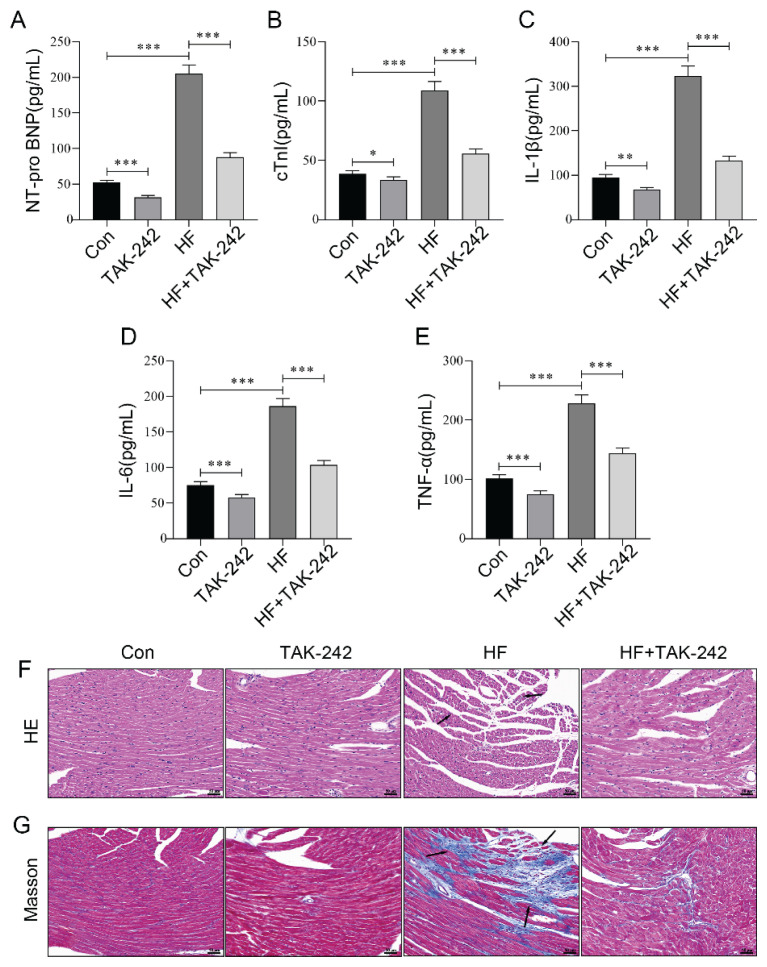
Inhibition of TLR4 attenuated myocardial injury in ISO-induced HF aged mice. (**A–E**) ELISA was employed to measure the serum concentrations of NT-proBNP, cTnI, IL-1β, IL-6, and TNF-α in HF aged mice following TLR4 inhibitor intervention. (**F**) HE staining was performed to assess the pathological changes in myocardial tissue of HF aged mice following TLR4 inhibitor intervention. Nuclei are stained blue and cytoplasm red. Arrows indicate pathological alterations such as myofiber rupture. Scale bar=50 μm. (**G**) Masson staining was conducted to evaluate collagen fiber deposition in the myocardial tissue of HF aged mice following TLR4 inhibitor intervention. Collagen fibers appear blue, while muscle fibers, fibrin, and red blood cells are stained red. Arrows highlight regions of excessive collagen accumulation. Scale bar = 50 μm. n=8, * p<0.05, ** p<0.01, *** p<0.001.

After confirming that inhibiting TLR4 could improve cardiac function in aged HF mice, we further investigated the associated indicators of myocardial injury. NT-proBNP, secreted by ventricular myocytes in response to increased ventricular wall tension, is negatively correlated with EF and serves as a reliable marker for detecting cardiac functional abnormalities such as elevated ventricular wall tension [29]. cTnI is specifically expressed in the thin filaments of cardiomyocyte myofibrils, plays a critical role in regulating myocardial contraction and directly reflects cardiomyocyte injury or necrosis [30]. These two biomarkers exhibit complementary diagnostic value in the clinical assessment of HF [31]. ELISA results demonstrated that ISO induction significantly increased serum levels of NT-proBNP, cTnI, and inflammatory factors such as IL-1β, IL-6, and TNF-α. Inhibition of TLR4 effectively reduced these markers (Fig. 3A–E). Moreover, HE and Masson staining revealed that in normal mouse myocardial tissue, myocardial fibers were tightly and orderly arranged, with no evident blue collagen deposition. Following ISO induction, cardio-myocyte volume increased, their arrangement became disordered, striations became blurred or disappeared, and there was excessive proliferation of collagen fibers in the myocardial interstitium, resulting in extensive blue collagen deposition. TLR4 inhibition markedly mitigated ISO-induced myocardial tissue damage (Fig. 3F–G). The aforementioned research findings further demonstrate that the inhibition of TLR4 can ameliorate HF in aged mice.

### Inhibition of TLR4 decreased NETs formation in aged mice with HF

**Fig. 4:**
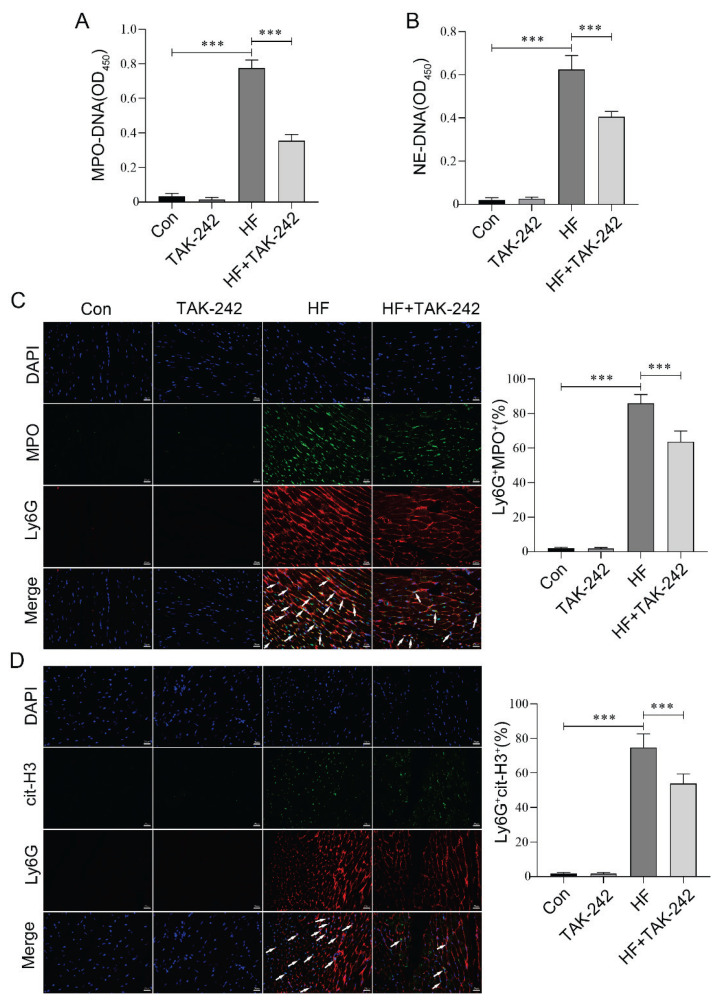
Inhibition of TLR4 decreased NETs formation in aged mice with HF. (**A–B**) ELISA was employed to quantify the levels of MPO-DNA and NE-DNA in the serum of HF aged mice following TLR4 inhibitor intervention. (**C–D**) Immunofluorescence staining was performed to examine the colocalization of Ly6G with MPO and Ly6G with cit-H3 in the myocardial tissue of HF aged mice following TLR4 inhibitor intervention. Arrows indicate co-localized cells. Scale bar = 20 μm. n=8, *** p<0.001.

TLR4 promotes the formation of NETs by interacting with pathways such as high-mobility group box 1 (HMGB1), thereby amplifying the neutrophil-mediated inflammatory response [32]. To investigate whether inhibiting TLR4 could suppress the formation of NETs, we examined the NETs markers in mice from each group. The ELISA results demonstrated that inhibiting TLR4 significantly reduced the levels of MPO-DNA and NE-DNA in the serum of ISO-induced aged HF mice (Fig. 4A–B). Furthermore, immunofluorescence results indicated that TLR4 inhibition reduced the co-localization level of Ly6G with either MPO or cit-H3 in the myocardial tissue of ISO-induced aged HF mice (Fig. 4C–D). These findings collectively suggest that inhibiting TLR4 effec-tively blocks the formation of NETs, and it may be an important related molecular mechanism.

### NETs inhibition alleviated ISO-induced HF in aged mice

**Fig. 5:**
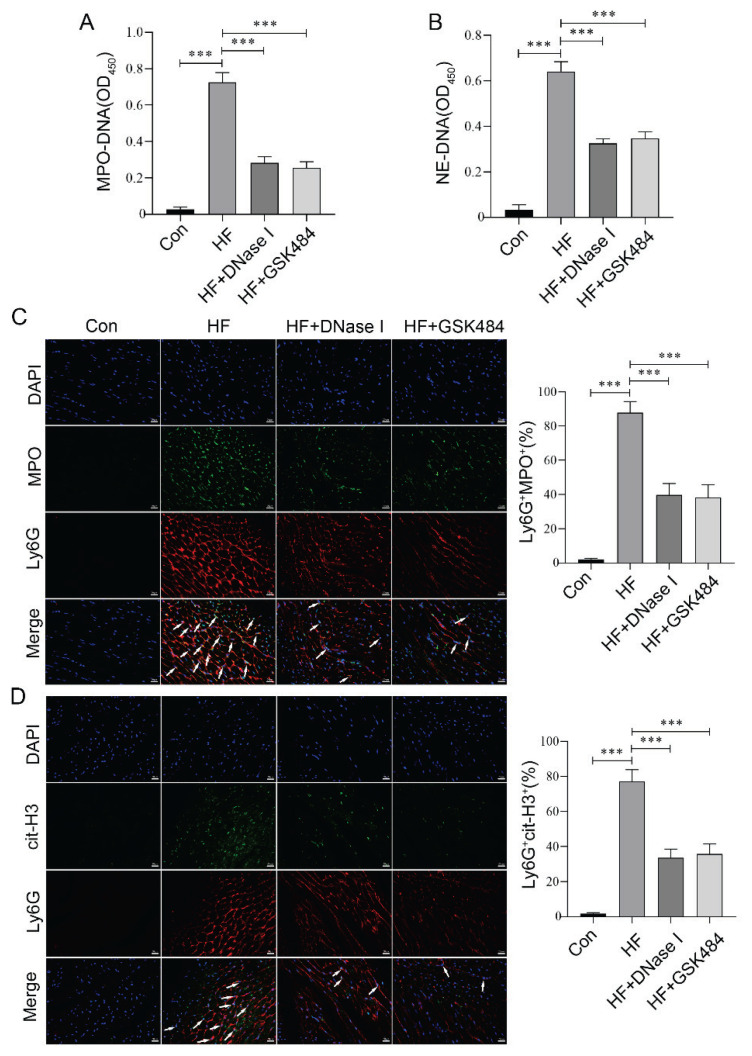
Inhibition of NETs formation was achieved using specific inhibitors. (**A–B**) ELISA was utilized to measure the levels of MPO-DNA and NE-DNA in the serum of HF aged mice after NETs inhibition. (**C–D**) Immunofluorescence staining was conducted to examine the co-localization of Ly6G with either MPO or cit-H3 in the myocardial tissue of HF aged mice after NETs inhibition. Arrows indicate co-localized cells. Scale bar = 20 μm. n=8, *** p<0.001.

**Fig. 6:**
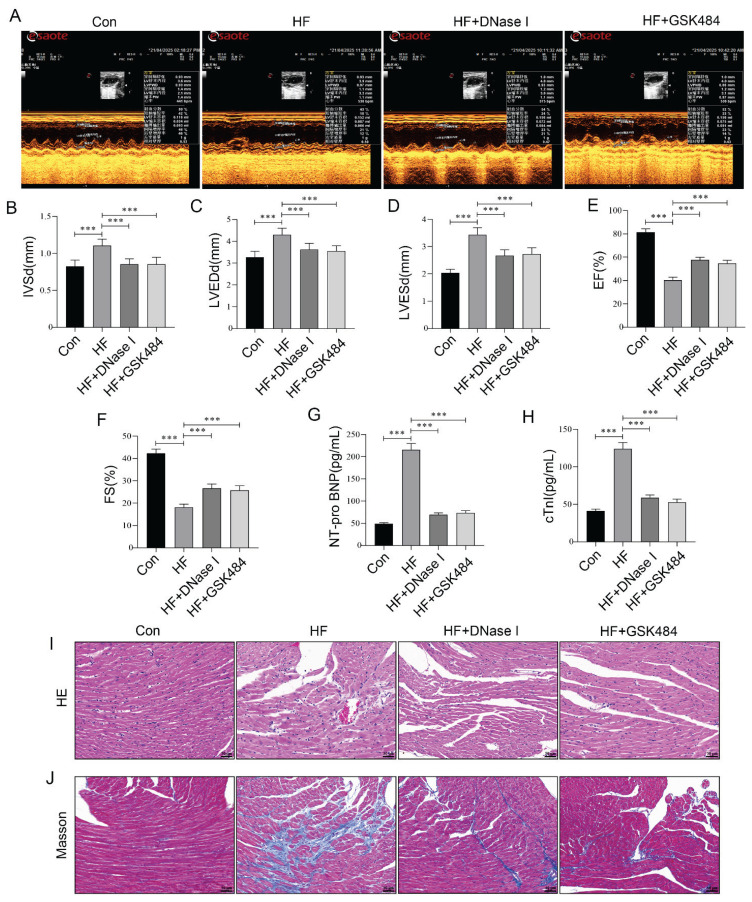
NETs inhibition alleviated ISO-induced HF in aged mice. (**A–F**) Changes in cardiac ultrasonic parameters across all groups of mice. (**G–H**) ELISA was employed to quantify the serum levels of NT-proBNP and cTnI in HF aged mice following NETs inhibition. (**I**) HE staining was performed to assess myocardial histopathological changes after NETs suppression. Nuclei are stained blue and cytoplasm red. Arrows indicate pathological alterations such as myofiber rupture. Scale bar = 50 μm. (**J**) Masson staining was used to evaluate collagen deposition in myocardial tissue post-NETs inhibition. Collagen fibers appear blue, whereas muscle fibers, fibrin, and red blood cells are stained red. Arrows highlight regions of excessive collagen accumulation. Scale bar = 50 μm. n=8, *** p<0.001.

DNase I and GSK484 are two key drugs that inhibit the formation of NETs or eliminate NETs *via* distinct mechanisms, demonstrating therapeutic potential in various disease models. DNase I, an endonuclease, directly degrades the DNA scaffold within NETs, thereby disrupting their structural integrity and mitigating their pathological effects [33]. In contrast, GSK484 acts as a PAD4 inhibitor, suppressing NETs formation by blocking citrullination of cit-H3 [34]. Previously, we confirmed that the down-regulation of TLR4 may improve HF in mice by inhibiting NETs formation. To further validate that suppression of NETs formation contributes to the improvement of HF, we administered DNase I and GSK484 in a mouse model of HF. Upon intervention with DNase I and GSK484 in HF mice, we observed a significant reduction in NETs formation in the myocardial tissue of mice, as evidenced by decreased levels of MPO-DNA and NE-DNA in serum (Fig. 5A–B), as well as reduced co-localization level of Ly6G with either MPO or cit-H3 in myocardial tissue (Fig. 5C–D). Moreover, inhibiting or eliminating NETs through these distinct pathways improved HF outcomes, characterized by enhanced cardiac function (Fig. 6A–F), reduced NT-proBNP and cTnI levels in serum (Fig. 6G–H), and alleviated pathological damage and collagen deposition in myocardial tissue (Fig. 6I–J). These findings align with the effects of TAK-242 intervention, suggesting that inhibiting TLR4 may ameliorate aged HF by reducing NETs formation.

## Discussion

As the terminal stage of cardiovascular disease, the progressive deterioration of cardiac function during the pathological process of HF ultimately results in irreversible myocardial damage. Elderly patients frequently encounter repeated hospitalizations and long-term care requirements due to multimorbidity, imposing significant economic burdens on families and society [35]. The current standardized treatment regimen relies on the “golden triangle” drugs (ACE inhibitors/angiotensin receptor blockers, β-blockers, aldosterone antagonists), supplemented with newer agents such as sacubitril/valsartan and dapagliflozin, which have been shown to markedly alleviate the clinical symptoms of HF. Nevertheless, the rates of drug utilization and achievement of therapeutic targets remain suboptimal in clinical practice [36,37]. Focusing on the molecular mechanisms underlying heart failure and identifying reversal targets for myocardial remodeling is crucial for enhancing patient prognosis and quality of life. In this study, we explored the role and underlying mechanisms of TLR4 in HF improvement and obtained the following findings: TLR4 inhibition significantly enhanced cardiac function, mitigated myocardial injury and fibrosis, and suppressed NETs formation in the myocardial tissue of HF mice. These results indicate that TLR4 inhibition may ameliorate HF by reducing NETs formation, thereby attenuating inflammatory-mediated damage.

The pathophysiological process of HF is intricately linked to elevated levels of systemic inflammatory factors. The underlying mechanism encompasses inflammation-mediated oxidative stress responses, mitochondrial dysfunction, and impaired regulation of calcium homeostasis, all of which culminate in myocardial dysfunction [38]. Research has demonstrated that patients with heart failure with reduced ejection fraction (HFrEF) exhibit a characteristic and significant increase in circulating inflammatory markers [39,40]. TLR4 is a pivotal factor in the inflammatory response, amplifying the inflammatory cascade *via* MyD88-dependent and TRIF-dependent signal transduction pathways, and playing a critical role in the onset and progression of cardiovascular diseases [41,42]. Notably, members of the TLR4 receptor family exhibit specific distribution patterns in myocardial tissue, with TLR4 being predominantly expressed in cardiac endothelial cells, smooth muscle cells, and cardio-myocytes. Among these, TLR4 demonstrates the highest expression abundance. Abnormal activation of TLR4 has been confirmed to contribute to the pathogenesis of major cardiovascular diseases, including myocarditis, acute myocardial infarction, atherosclerosis, and heart failure [43–45]. Our findings demonstrate that TLR4 inhibition reduces IVSd, LVEDd, and LVESd while increasing FS and EF in HF mice. Concurrently, myocardial pathological damage is attenuated and collagen fiber deposition is diminished. These results are consistent with the report by Li *et al.* that activation of the TLR4/NF-κB pathway exacerbates cardiac dysfunction [46]; however, our study extends these observations by providing direct interventional evidence for the therapeutic potential of suppressing this signaling axis. Notably, TLR4 inhibition significantly reduced serum levels of cardiac injury biomarkers NT-proBNP and cTnI in HF mice, along with marked decreases in pro-inflammatory cytokines including IL-1β, IL-6, and TNF﷓α. This establishes a more direct link between TLR4 suppression and the core inflammatory pathophysiology of HF. Furthermore, our data expand upon the work of Qi *et al.*, who identified the RTN3-HSPB1-TLR4 axis as a driver of mitochondrial dysfunction, inflammation, and myocardial infarction, by demonstrating its broader implications in systemic inflammation and myocardial injury [47]. Collectively, these results highlight the beneficial effects of targeting TLR4 on both inflammatory responses and cardiac damage markers in an animal model of HF.

Neutrophils serve as the first line of immune defense and contribute to host protection through phagocytosis and sterilization. Upon encountering macromolecular pathogens such as fungal hyphae and parasite cysts, neutrophils release a network structure known as NETs, which consist of a DNA scaffold, cit-H3, and MPO and NE *via* the NETosis process [48,49]. Notably, during myocardial injury, neutrophils exhibit an N1 pro-inflammatory phenotype, significantly enhancing their activity and accelerating NETs formation [50,51]. However, excessive NETs release not only provides a scaffold for platelet aggregation but also activates the coagulation cascade, stimulates endothelial cells to release procoagulant factors, and amplifies the coagulation response, thereby increasing the risk of thrombosis and microcirculatory dysfunction [52–54]. Consequently, strategies targeting the regulation of NETs generation (e.g., inhibition of PAD4 enzyme activity) or degradation (e.g., DNase I treatment) may offer novel therapeutic approaches to improve the prognosis of elderly HF patients. Accumulating evidence indicates that TLR4 promotes NETs formation through multiple signaling pathways [55,56]. Given this, we investigated the relationship between TLR4 and NETs – an emerging contributor to disease pathogenesis. Our results demonstrate that TLR4 inhibition significantly reduces serum levels of MPO-DNA and NE-DNA in HF mice and decreases co-localization of Ly6G with MPO or cit-H3 in myocardial tissue, indicating suppressed NETosis. This aligns with previous findings showing that the HMGB1-TLR4 positive feedback loop amplifies NETs generation in liver ischemia-reperfusion injury [57], but our study is the first to systematically establish TLR4 as a critical driver of NETs formation in the context of HF. To further validate the functional involvement of NETs, we employed two distinct interventions – DNase I (to degrade existing NETs) and GSK484 (a selective PAD4 inhibitor that blocks NETs formation) – and observed that both recapitulated the cardioprotective effects of TLR4 inhibition. These findings position TLR4 as an upstream regulatory hub in NETs-driven inflammation in HF and provide novel insights into the immunopathological mechanisms underlying heart failure.

In conclusion, this study demonstrated that inhibiting TLR4 can enhance cardiac function and reverse myocardial tissue damage in HF mice. The underlying mechanism may involve blocking the formation of NETs and modulating excessive inflammatory responses. This finding offers a promising strategy for improving cardiac function, quality of life, and health prognosis in elderly patients with cardiovascular diseases. Nevertheless, given the intricate pathological mechanisms of heart failure, further investigation is warranted to elucidate the key molecular events and regulatory mechanisms upstream and downstream of the TLR4 signaling network. Specifically, understanding the interaction between NETs-mediated inflammatory cascades and myocardial remodeling will provide a robust scientific basis for the clinical translation and application of TLR4 inhibitors.
